# Normal ventral telencephalic expression of *Pax6 *is required for normal development of thalamocortical axons in embryonic mice

**DOI:** 10.1186/1749-8104-4-19

**Published:** 2009-06-05

**Authors:** T Ian Simpson, Thomas Pratt, John O Mason, David J Price

**Affiliations:** 1 Genes and Development Group, Centre for Integrative Physiology, University of Edinburgh, Hugh Robson Building, George Square, Edinburgh, EH8 9XD, UK

## Abstract

**Background:**

In addition to its well-known expression in dorsal telencephalic progenitor cells, where it regulates cell proliferation and identity, the transcription factor Pax6 is expressed in some ventral telencephalic cells, including many postmitotic neurons. Its functions in these cells are unknown.

**Results:**

We generated a new floxed allele of *Pax6 *and tested the consequences of a highly specific ventral telencephalic depletion of Pax6. We used the *Six3*^*A1A2*^-*Cre *allele that drives production of Cre recombinase in a specific region of Pax6-expression close to the internal capsule, through which thalamic axons navigate to cerebral cortex. Depletion in this region caused many thalamic axons to take aberrant routes, either failing to turn normally into ventral telencephalon to form the internal capsule or exiting the developing internal capsule ventrally. We tested whether these defects might have resulted from abnormalities of two structural features proposed to guide thalamic axons into and through the developing internal capsule. First, we looked for the early pioneer axons that project from the region of the future internal capsule to the thalamus and are thought to guide thalamocortical axons to the internal capsule: we found that they are present in conditional mutants. Second, we examined the development of the corridor of Islet1-expressing cells that guides thalamic axons through ventral telencephalon and found that it was broader and less dense than normal in conditional mutants. We also examined corticofugal axons that are thought to interact with ascending thalamocortical axons, resulting in each set providing guidance to the other, and found that some are misrouted to lateral telencephalon.

**Conclusion:**

These findings indicate that ventral telencephalic Pax6 is important for formation of the Islet1-expressing corridor and the thalamic and cortical axons that grow through it. We suggest that Pax6 might affect thalamic axonal growth indirectly via its effect on the corridor.

## Background

The cerebral cortex receives most of its sensory innervation via thalamocortical axons, which start to form at about embryonic day 12.5 (E12.5) in mice. Thalamic axons initially grow antero-ventrally through the diencephalon before turning sharply laterally, avoiding ventral diencephalic regions containing the hypothalamus and forming the internal capsule in ventral telencephalon. After exiting the internal capsule, axons turn dorsally to reach cortex from about E13.5 [1,2]. Previous studies have indicated two major mechanisms likely to guide advancing thalamic growth cones into and through ventral telencephalon [3-9]. First, several studies have suggested that a transient group of ventral telencephalic neurons project pioneer axons to the thalamus by E12.5, providing guidance for reciprocal thalamocortical axons [4,6,8-14]. Second, work by Lopez-Bendito *et al*. [15] showed that cells migrate from the lateral ganglionic eminence (LGE) to form a ventral telencephalic permissive corridor marked by expression of the transcription factor Islet1, through which thalamocortical axons grow.

Many transcription factors well-known for their functions in early patterning of the developing nervous system also have important functions in subsequent regulation of axonal navigation, by influencing the responses of growing axons and/or the deployment of guidance cues [16-18]. Pax6 is one such factor. In mice lacking Pax6, thalamic axons fail to navigate correctly in the ventral telencephalon and a normal internal capsule does not form [12,19-21]. The mechanism of action of Pax6 in thalamocortical axonal development is unknown.

A critical step towards understanding how Pax6 regulates thalamocortical development is to define its site(s) of action. Comparing Pax6's spatio-temporal pattern of expression with the timetable of thalamocortical tract formation indicates that Pax6 could influence guidance by actions at the origin of the tract and/or in its target and/or in intermediate tissue [1,2,4-6,19,22-24]. Pax6 is expressed in the embryonic thalamus before thalamic axons develop [19,20,23-28]. Previous experiments using a co-culture approach have indicated that Pax6 is required in the thalamus for thalamocortical axons to navigate through ventral telencephalon [20]. Pax6 is expressed in the cerebral cortex from before the time when thalamic axons reach it [27,29,30]. A recent study by Pinon *et al. *[31] reported normal thalamocortical tract development following targeted deletion of *Pax6 *specifically in cerebral cortex from before the time of thalamocortical development, suggesting that Pax6 is not required in the cortex for thalamic axonal guidance. Pax6 is expressed by some ventral telencephalic cells in the vicinity of the internal capsule at the time when thalamocortical axons are navigating through this intermediate region [19,27,32,33]. Its functions in these ventral cells are unknown; a logical extension of previous work is to investigate whether Pax6 in this region contributes to the generation of normal thalamocortical projections and, if so, how. These issues form the focus of the present study.

We addressed the possibility that normal expression of Pax6 in ventral telencephalic cells around the internal capsule is required for normal thalamocortical development. We generated a new floxed allele of *Pax6 *and crossed it with a strain of mice in which Cre recombinase expression is restricted to a very specific Pax6-expressing region of ventral telencephalon around the future internal capsule from before E12.5. This resulted in an early depletion of Pax6-expressing cells in this region and caused many thalamic axons to take aberrant routes. We tested possible mechanisms for these axonal defects and found that the Islet1-expressing corridor that normally guides thalamocortical axons failed to develop normally in conditional mutants.

## Results

### Generation of the floxed *Pax6 *allele

**Figure 1:**
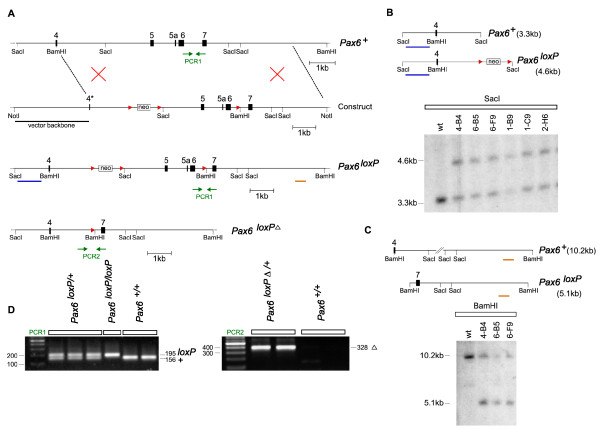
**Generation of the *Pax6 *conditional allele**. **(A) **Targeting strategy showing the insertion of *loxP *sites into introns 4 and 6 of murine *Pax6*, flanking exons that encode the paired type homeodomain. The final floxed *Pax6 *(*Pax6*^*loxP*^) and floxed null (*Pax6*^*loxPΔ*^) alleles are shown. **(B, C) **Correct targeting of the *Pax6 *locus in a series of embryonic stem cell clones was confirmed by Southern blot hybridization with probes (indicated by colored bars) proximal (B) and distal (C) to the insertion site; wt = wild type. **(D) **PCR amplification was used to genotype the *Pax6*^*loxP *^allele using primer set PCR1 and *Pax6*^*loxPΔ *^alleles using primer set PCR2 (positions of primers are indicated in (A)).

In the floxed allele of *Pax6 *(Figure 1A), *loxP *sites flank exons 5, 5a and 6, which encode the paired domain and are essential for Pax6 function [34]. Cre-mediated deletion of these exons was predicted to cause a nonsense frame-shift in the remaining transcript, which includes sequences encoding the second DNA binding domain, the Pax6 homeodomain. As the *loxP *sites were inserted into non-conserved intronic regions of the *Pax6 *gene the floxed allele was predicted to function normally. *Pax6*^*loxP/loxP *^mice are viable, fertile and phenotypically indistinguishable from wild-type mice (n > 100), both on gross morphology and in sectioned material, and express Pax6 normally (Additional file 1A-B").

To establish that the *Pax6*^*loxP *^allele could generate a phenotype similar to that caused by the commonly used *Pax6*^*SeyEd *^loss-of-function allele, we used mice that express Cre recombinase in oocytes under the control of the *Zp3 *(zona pellucida glycoprotein 3) promoter to generate a line carrying the deleted allele, *Pax6*^*loxPΔ *^[35]. *Pax6*^*loxPΔ/loxPΔ *^embryos were negative for Pax6 protein as determined by immunohistochemistry with antibodies that recognize epitopes encoded within or 3' to the paired domain [36] (Additional file 1C', C"). *Pax6*^*SeyEd/SeyEd *^embryos showed very faint immunoreactivity for Pax6 (Additional file 1D', D"), most likely because the antibodies' epitopes are 5' to the mutation and could be included in a truncated protein present at low level. Both *Pax6*^*loxPΔ/loxPΔ *^and *Pax6*^*SeyEd/SeyEd *^embryos had similar severe defects of the eyes and face [37,38] (data not shown) and telencephalon (Additional file 1C, D).

### Generating a restricted loss of Pax6 expression in ventral telencephalon

In the *Six3*^*A1A2*^-*Cre *transgenic mouse, Cre recombinase is expressed in ventral telencephalon but in neither thalamus nor dorsal telencephalon [39]. The details of its expression in ventral telencephalon were unknown and so we crossed *Six3*^*A1A2*^-*Cre *mice to the reporter line *Z/AP *in which human placental alkaline phosphatase (hPLAP) is expressed in cells carrying Cre-mediated recombination [40]. We studied expression of hPLAP at the time when thalamic axons are first navigating through ventral telencephalon to cortex, E12.5 to E14.5.

**Figure 2:**
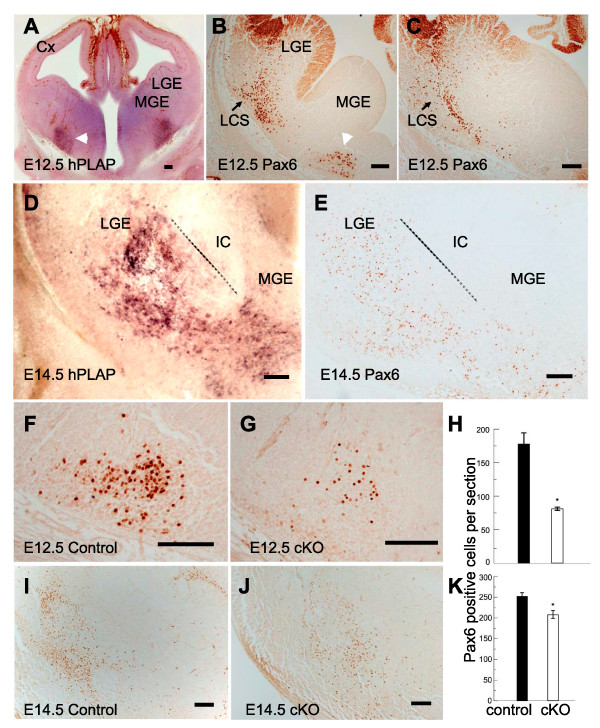
**Depletion of ventral telencephalic Pax6 using the *Six3*^*A1A2*^-*Cre *allele**. **(A, D) **The *Z/AP *allele was used to reveal sites of Cre activity at E12.5 and E14.5 by expression of human placental alkaline phosphatase (hPLAP). Cre activity was restricted to bilateral patches in the ventral telencephalon (for example, white arrowhead in (A): note that brown stain elsewhere in lines of cells running throughout the tissue and along the pial surface is alkaline phosphatase reactivity in blood vessels). **(B, C, E) **Immunohistochemistry for Pax6 expression in the ventral telencephalon of normal embryos aged E12.5 and E14.5; the section in (B) is rostral to that in (C); the white arrowhead in (B) indicates a cluster of Pax6-expressing cells in the ventral medial ganglionic eminence (MGE) in a similar position to the hPLAP staining marked by the white arrowhead in (A); the section in (E) is of the same region as that shown in (D). Broken lines in (D, E) mark the internal capsule's ventral edge. **(F, G) **Immunohistochemistry for Pax6 expression in the region indicated by the white arrowhead in (B) in control and conditional knockout (cKO) embryos at E12.5; quantification of the reduced density of Pax6-expressing cells in this region is shown in **(H)**. **(I, J) **Immunohistochemistry for expression of Pax6 in the region shown in (D, E) in control and cKO embryos at E14.5; quantification of the reduced density of Pax6-expressing cells in this region is shown in **(K)**. Abbreviations: Cx, cortex; IC, internal capsule; LCS, lateral cortical stream; LGE, lateral ganglionic eminence. Scale bars: 50 μm.

At E12.5, hPLAP-expressing cells were detected in a ventral domain of the medial ganglionic eminence (MGE; Figure 2A, white arrowhead), close to the region where thalamic axons turn into the ventral telencephalon to form the internal capsule. A cluster of Pax6-expressing cells was located in ventral MGE in a similar position to the hPLAP staining (Figure 2B, white arrowhead). This was separate from a more lateral population of Pax6-expressing cells derived from the pallium and forming part of the lateral cortical stream (LCS) [41] (Figure 2B, C, black arrows). The LCS comprises pallial- and subpallial-derived neural progenitor cells derived from the pallial-subpallial border (PSPB) that migrate to the developing structures of the ventral telencephalon, including the piriform cortex and amygdala. By E14.5, hPLAP-expressing cells were detected more widely throughout not only the MGE but also the LGE, still mainly ventral to the internal capsule (Figure 2D) in a region containing many Pax6-expressing cells (Figure 2E). Based on these comparisons, we predicted that the *Six3*^*A1A2*^-*Cre *transgene would produce a very specific loss of Pax6 expression from *Pax6*^*loxP/loxP *^mice, confined to a region ventral to the developing internal capsule.

We quantified the loss of Pax6-expressing cells in the ventral telencephalic regions where *Six3*^*A1A2*^-*Cre *was expressed at E12.5 and E14.5 in controls and conditional knockouts (cKOs). We calculated the average number of Pax6-expressing cells per section in the ventral MGE at E12.5 and ventral MGE and LGE at E14.5 for cKOs and controls (n = 4 embryos in each group). In E12.5 cKOs, there was a greater than 50% reduction in the density of Pax6-expressing cells in the ventral domain of the MGE close to the region where thalamic axons turn into the ventral telencephalon to form the internal capsule (Figure 2F–H; *P *< 0.01, Student's *t*-test). There was also a significantly decreased density of Pax6-expressing cells in ventral MGE and LGE in older E14.5 cKOs (Figure 2I–K; *P *< 0.03, Student's *t*-test), although the difference was smaller than at E12.5. The reason for this is most likely the continued influx of non-deleted Pax6-expressing cells via the LCS from more lateral telencephalic regions around the PSPB [41,42] where *Six3*^*A1A2*^-*Cre *is not active. For the present study, our important finding was that cKO E12.5 to 14.5 embryos have a depleted population of Pax6-expressing cells ventral to the developing internal capsule at the time when it starts to form. As anticipated, expression of Pax6 in the cortex and thalamus was unaffected in cKOs (Additional file 2).

### Depletion of Pax6 in ventral telencephalon disrupts thalamocortical axonal development

To determine whether depletion of Pax6 in the ventral telencephalon affected thalamocortical development, we injected crystals of DiI into the dorsal thalamus at E12.5, E14.5 and E16.5 (n ≥ 4 embryos in all groups; reported differences between control and cKO embryos were consistent in all embryos).

**Figure 3:**
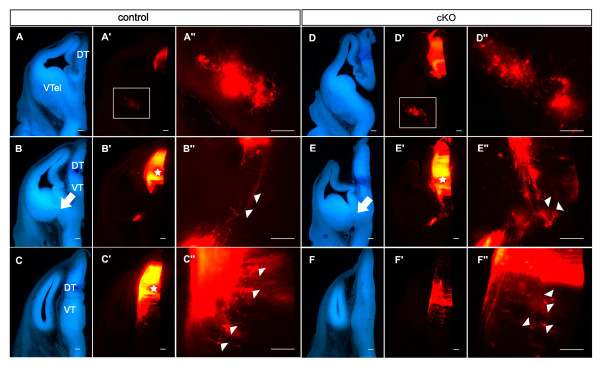
**Disruption of thalamic axons in E12.5 *Pax6 *conditional knockouts**. A rostral-to-caudal series of coronal 100 μm vibratome sections is shown for control and conditional knockout (cKO) embryos. **(A-F) **Bisbenzimide staining; (A', A"-F', F") corresponding low and high magnification views of DiI labeling from the dorsal thalamus (DT). Stars in (B', C', E') indicate the points of DiI crystal placement. Boxed areas in (A', D'), shown in (A", D"), respectively, demonstrate that retrogradely labeled neurons are present in ventral telencephalon (VTel) in both control and cKO embryos. In control embryos the axons from dorsal thalamus are oriented towards and intermingle neatly with the axons of ventral telencephalic neurons (white arrowheads in (B")); in cKOs, many thalamic axons are disorganized in their projection (white arrowheads in (E")); the white arrows in (B, E) indicate the centers of the regions shown in (B", E"). Both control and cKO embryos have retrogradely labeled cell bodies in the ventral thalamus (VT) (white arrowheads in (C", F")). Scale bars: 50 μm.

As previously described [5,10,43], injections into the dorsal thalamus of E12.5 control embryos retrogradely labeled a population of cell bodies in the ventral part of the MGE (Figure 3A-A"). The axons from these cells mingle with those exiting the dorsal thalamus and are postulated to guide them as they traverse the ventral thalamus towards the diencephalic-telencephalic border [44,45]. Figure 3B-B" shows a plane of section adjacent to the main bundle of overlapping reciprocal projections, allowing clear visualization of the trajectories of the two sets of axons, which were oriented directly towards each other (white arrowheads in Figure 3B"; the white arrow in Figure 3B indicates the region at the diencephalic-telencephalic border shown at higher magnification in Figure 3B"). In addition, retrogradely labeled cell bodies were seen in the ventral thalamus at this age (Figure 3C-C"). Injection of DiI into the dorsal thalamus of E12.5 cKO mice also retrogradely labeled cell bodies in the ventral MGE (Figure 3D-D"). There were abnormalities in the trajectories of axons from the dorsal thalamus (Figure 3E-E"): many did not follow a normal turn towards the ventral telencephalon (for example, white arrowheads in Figure 3E", compare with Figure 3B"; the white arrow in Figure 3E indicates the region at the diencephalic-telencephalic border shown at higher magnification in Figure 3E"). As in controls, retrogradely labeled cell bodies were observed in the ventral thalamus of cKO mice (Figure 3F-F").

**Figure 4:**
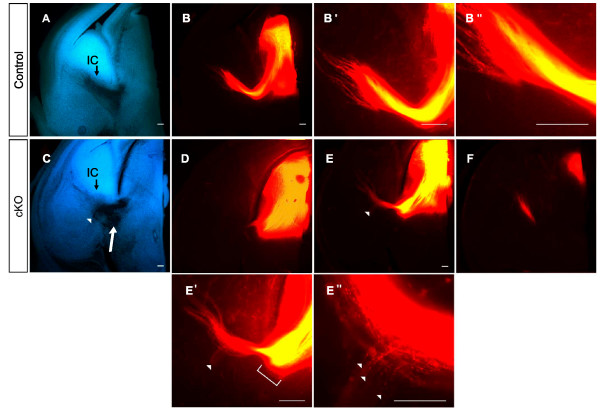
**Disruption of thalamic axons in E14.5 *Pax6 *conditional knockouts**. Coronal vibratome sections (200 μm) for control and conditional knockout (cKO) embryos injected with DiI in the dorsal thalamus: **(A, C) **bisbenzimide stained sections from (A) control and (C) cKO; **(B-B") **DiI labeling from control at increasing magnifications; **(D-F) **serial sections from cKO with central section (E) shown at increasing magnifications in (E', E"). In control embryos, the dense bundle of thalamic axons traversing the diencephalon turns into the ventral telencephalon and travels through the internal capsule (IC). In cKOs, although a dense normal-appearing bundle of thalamic axons traverses the diencephalon (E), much of the bundle appears to end at the diencephalic-telencephalic border (bracket in (E')), fewer thalamic axons traverse the internal capsule and some of those axons that do penetrate the internal capsule exit it ventrally (at the position of the white arrowhead in (C); labeled axons exiting ventrally are indicated by white arrowheads in (E-E")). The white arrow in (C) indicates the diencephalic-telencephalic border. Scale bars: 50 μm.

In E14.5 controls, the internal capsule was visible in bisbenzimide stained sections as a cell sparse area (Figure 4A) that corresponded with the tightly fasciculated bundle of thalamocortical axons labeled by DiI from the dorsal thalamus (Figure 4B). This bundle showed evidence of defasciculation as it emerged from the internal capsule but not before (Figure 4B'-B"). In E14.5 cKO mice, the overall shape of the internal capsule was strikingly different in bisbenzimide stained sections: the cell-sparse zone spanning the diencephalic-telencephalic border appeared disorganized (white arrow in Figure 4C) and only a narrow cell-sparse shaft extended across the ventral telencephalon (black arrow in Figure 4C; compare with Figure 4A). DiI tracing from the thalamus revealed that, while the bundles of thalamic axons descending towards the diencephalic-telencephalic boundary in cKOs were similar in size to those in controls, an abnormally thin bundle of thalamocortical axons crossed the ventral telencephalon, suggesting that many failed to turn into the internal capsule (Figure 4D–F). Much of the bundle of axons descending through the diencephalon appeared to end at the diencephalic-telencephalic border (bracket in Figure 4E'). In addition, small numbers of axons left the internal capsule along its length (in the region indicated by the white arrowhead in Figure 4C) to grow in a ventral direction (white arrowheads in Figure 4E'-E").

**Figure 5:**
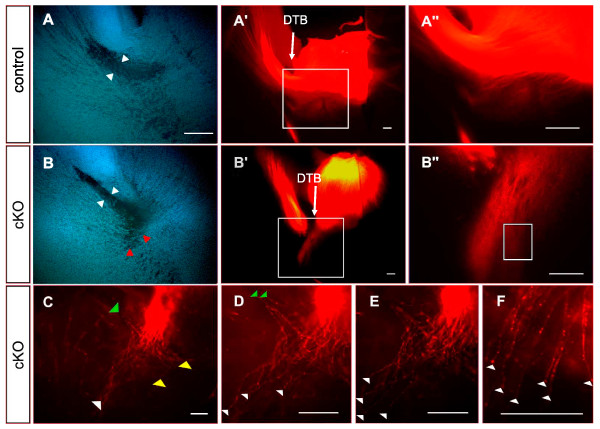
**Disruption of thalamic axons in E16.5 *Pax6 *conditional knockouts**. Coronal vibratome sections for control and conditional knockout (cKO) embryos injected with DiI in the dorsal thalamus. **(A, B) **Bisbenzimide stained sections show narrowing of the internal capsule between the white arrowheads. **(A'-B") **Corresponding DiI labeled sections; boxed areas in (A', B') are shown in (A", B"), respectively. In cKOs, many thalamic axons do not turn across the diencephalic-telencephalic border (DTB) but descend ventrally towards the hypothalamus. This aberrant tract corresponds to a cell-free spur seen in bisbenzimide-stained sections near the start of the internal capsule (red arrowheads in (B)). **(C-F) **High magnification optical sections from around the boxed area in (B"): many aberrant axons descend directly ventrally (white arrowheads) while a few loop out medially (yellow arrowheads) or project laterally into ventral telencephalon (green arrowheads). Scale bars: 50 μm.

As at E14.5, the internal capsule of E16.5 cKO mice appeared abnormally narrow (Figure 5A, B, between white arrowheads). We confirmed that the overall size of the internal capsule was reduced in E16.5 cKOs by summing the surface areas of the cell-sparse regions in serial bisbenzimide-stained sections from each of three control and three cKO embryos: values were significantly smaller in cKO embryos (cKO, 3,791 ± 157 (standard error of the mean) μm^2^; control, 5,180 ± 259 μm^2^; *P *< 0.02, Student's *t*-test).

In E16.5 controls, DiI injections into the dorsal thalamus labeled a thick fascicle of axons exiting the diencephalon, turning to form the internal capsule and showing defasciculation on exiting the internal capsule (Figure 5A', A"). In contrast, DiI injections into the dorsal thalamus of cKO mice labeled a bifurcated tract in which some axons projected along a normal route but others coursed ventrally, in the direction of the hypothalamus, from the region around which the normal tract bent sharply to cross the diencephalic-telencephalic border (Figure 5B', B"). This abnormality corresponded with a cell-sparse region ventral to the bend of the tract at the diencephalic-telencephalic border seen in bisbenzimide-stained sections (Figure 5B, red arrowheads). Most of these ventral projections continued roughly parallel with the diencephalic-telencephalic border (Figure 5C–F, white arrowheads); a few reached the ventral surface of the brain but many extended no more than about 50 to 100 μm and ended with spots of intense fluorescence that were probably growth cones (for example, Figure 5F, left arrowhead). A few turned either laterally (Figure 5C, D, green arrowheads) or medially (Figure 5C, yellow arrowheads).

In summary, our findings indicate that depletion of Pax6 from ventral telencephalic cells around the future and developing internal capsule results in: the failure of many thalamic axons to navigate normally from the vicinity of the diencephalic-telencephalic border into the internal capsule; and the exit of some thalamic axons from the internal capsule in a ventral direction.

### Possible mechanisms of action of ventral telencephalic Pax6 in thalamic axonal guidance

Previous studies have proposed two major mechanisms guiding thalamic axons into the ventral telencephalon to form the internal capsule. First, a transient group of ventral telencephalic neurons projecting from the vicinity of the future internal capsule to the thalamus might provide guidance for thalamocortical axons [6,8-10,43]. Second, a set of Islet1-expressing cells derived from the LGE migrates to form a permissive corridor in the ventral telencephalon, through which thalamocortical axons grow forming the internal capsule [15].

Work described above showed that the transient group of ventral telencephalic neurons projecting to the thalamus was present at an appropriate age in cKOs. We assessed the size of the group. Counting individual neurons was not sufficiently accurate since the DiI labeling was cytoplasmic and the cells were so densely packed with numerous processes that they could not be resolved. We counted all labeled pixels above a threshold (set to remove background) from each of a series of sections through these groups of cells in four cKO and three control embryos. This provided, for each embryo, a measure of the total area of the retrogradely labeled cells, which we assume to be proportionate to the number of cells since cell sizes appeared similar in the two genotypes. We found no significant difference in the average areas between the two genotypes (control, 621 ± 146 (standard error of the mean) μm^2^, n = 3; cKO, 493 ± 114 μm^2^, n = 4; *P *= 0.51, Student's *t*-test).

**Figure 6:**
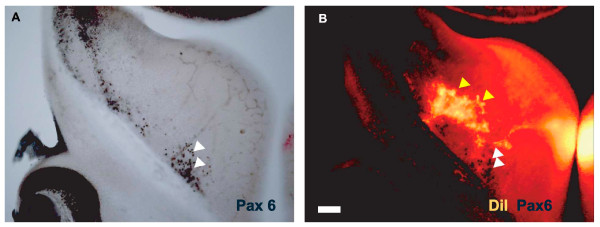
**Ventral telencephalic pioneer neurons do not express Pax6**. **(A, B) **Coronal vibratome sections from E12.5 control mice injected with DiI in the dorsal thalamus. After DiI diffusion, immunohistochemistry revealed cells expressing Pax6 (the most medial group is indicated by white arrowheads). (B) Under epifluoresence, the location of DiI retrogradely labeled cell bodies in the ventral telencephalon can be seen (yellow arrowheads). Retrogradely labeled cells do not express Pax6. Scale bar: 50 μm.

It remains possible that these pioneer neurons have molecular defects preventing their normal guidance of thalamic axons. The likelihood of this would have been enhanced if these transient projections normally expressed Pax6. To address this possibility, we combined immunohistochemistry for Pax6 with DiI labeling of control brains at E12.5. We found no overlap between the populations of cells expressing Pax6 in the ventral MGE (Figure 6A, B; the population labeled with white arrowheads corresponds to that indicated by the white arrowhead in Figure 2B) and projecting to the thalamus (Figure 6B, yellow arrowheads). This suggests that if these projections in cKOs do have molecular defects preventing them fulfilling their guidance role, it would have had to have been generated either indirectly, as a result of loss of Pax6 in other cells, or as a consequence of Pax6 loss earlier in these cells' lineage (assuming that they did express Pax6 earlier and that *Six3*^*A1A2*^-*Cre *caused loss of any such expression, which are formal but uncertain possibilities).

**Figure 7:**
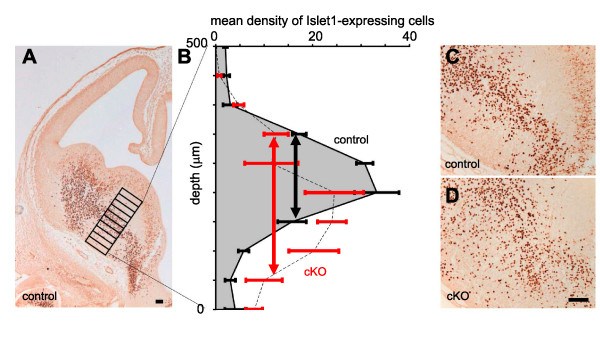
**Broadening of the Islet-1-expressing corridor in E12.5 conditional knockouts**. **(A) **Coronal section of the telencephalon of an E12.5 control embryo showing immunohistochemistry for Islet1; the arrangement of bins used for counting Islet1-expressing cells across the corridor in control and conditional knockout (cKO) embryos is marked. **(B) **Graph shows the distributions of Islet1-expressing cells across the corridor: values plotted are mean densities per bin ± standard error of the mean (n = 4 embryos for both control and cKO data); black error bars and grey shading are for control data; red error bars and broken line without shading are for cKO data; double-headed arrows indicate the widths of the distributions at half their peak values (black arrow for control data, red arrow for cKO data). **(C, D) **Illustration of the finding: in cKO embryos Islet1-expressing cells spread more broadly from the corridor towards the ventral surface of the brain (at the bottom left of each panel) than in controls. Scale bars: 50 μm.

A more parsimonious explanation for the defective formation of the internal capsule in cKOs came from an analysis of the permissive corridor in the ventral telencephalon of cKOs. In E12.5 control embryos we observed the previously described distribution of Islet1-expressing cells densely corralled into a well-defined corridor traversing the mantle zone of the MGE (Figure 7A, C). In cKO mice, Islet1-expressing cells were also distributed in a corridor running in a similar position through the MGE but they appeared more broadly distributed (Figure 7D). This difference was quantified in four control and four cKO E12.5 embryos. A series of sampling bins was laid across the centre of the corridor, starting from the pial surface (as shown in Figure 7A), in two non-adjacent sections from each embryo. Numbers of Islet1-expressing cells at each depth from each embryo were averaged to give the density profiles shown in Figure 7B. Although the overall numbers of Islet1-expressing cells in the entire sampling area were no different in controls and cKOs (mean for controls, 117.0 cells per 10 bins; mean for cKOs, 116.2 cells per 10 bins), the peak densities seen in the center of the corridor were lower in cKOs. The distribution of Islet1-expressing cells was broader in the ventral direction in cKOs: double-headed arrows in Figure 7B indicate the widths of the distributions at half peak values. These data suggest that normal numbers of Islet1-expressing cells migrate from the LGE but they are more scattered into ventral MGE in cKOs, where Pax6-expressing cells are depleted. This disruption of the corridor might account for the thalamic axonal navigation defects observed in cKOs.

### Evidence for aberrant corticofugal axons in cKOs

**Figure 8:**
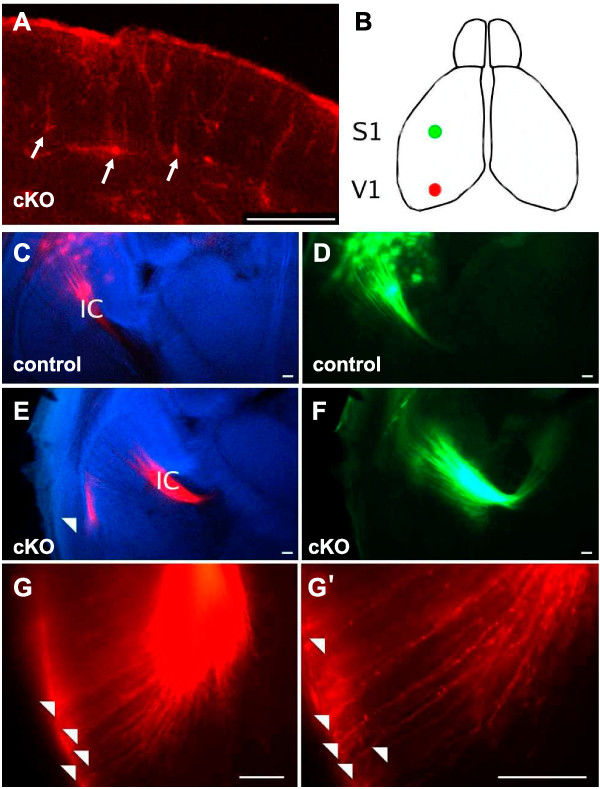
**Aberrant corticothalamic projections in conditional knockouts**. **(A) **Coronal section of E14.5 conditional knockout (cKO) cortex following injection of DiI in dorsal thalamus: retrogradely labeled cell bodies are visible, some are indicated by arrows. **(B) **Diagram of the forebrain showing sites of cortical injection of DiI (red in visual area 1 (V1)) and DiA (green in somatosensory area 1 (S1)). **(C, D) **Control E16.5 brains showing (C) DiI and (D) DiA label in the internal capsule (IC). **(E, F) **E16.5 cKO brains showing (E) DiI and (F) DiA label in the internal capsule; some aberrant DiI label from V1 was also seen traveling laterally (white arrowhead in (E)). **(G, G') **E16.5 cKO brains showing higher magnification of area indicted by the white arrowhead in (E): many labeled axons (arrowheads) are seen projecting to the pial surface. Scale bars: 50 μm.

Previous work has shown that ascending thalamocortical axons intermingle with descending corticofugal axons in the ventral telencephalon and this observation has led to the suggestion that interactions between the two sets of axons might be important for the guidance of each set [3,4,21]. Since in cKOs some thalamic axons are derailed before they reach the region ventral to the PSPB where they would interact with descending corticofugal axons, it seemed possible that an interaction between the populations of thalamocortical and corticofugal axons might be suboptimal, resulting in some descending cortical axons taking aberrant trajectories. Following injections of DiI into the thalamus of E14.5 and E16.5 cKOs, we observed retrogradely labeled cell bodies in the cortex (Figure 8A), indicating that corticothalamic projections are present. We then injected DiI and DiA into the cortex at E16.5, as shown in Figure 8B: this would result in both the retrograde labeling of thalamocortical axons and the anterograde labeling of corticofugal axons, thereby allowing any aberrantly routed corticofugal trajectories to be identified. Labeling of the axons between somatosensory cortex and thalamus appeared normal (Figure 8D, F) but, while we observed many axons labeled from the visual cortex in the internal capsule of both controls and cKOs (Figure 8C, E), some were seen in the lateral telencephalon of cKOs (Figure 8E, G, G') but not of controls (Figure 8C). These axons could be followed to the pial surface of the lateral telencephalon (Figure 8G, G') but not beyond. The likely explanation of this finding is that some descending corticofugal axons from visual cortex become misrouted in cKOs.

## Discussion

**Figure 9:**
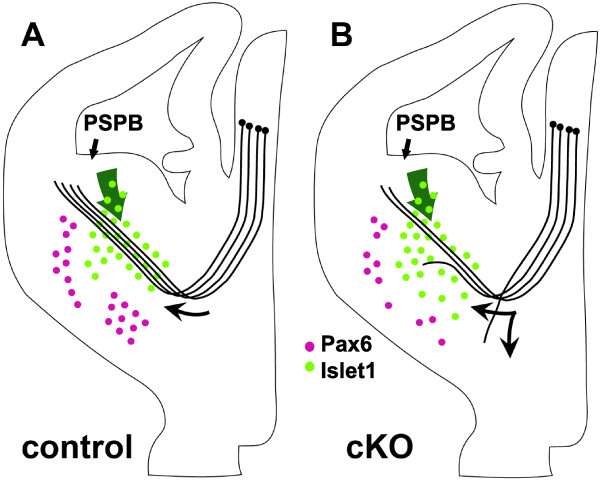
**Summary of findings**. **(A) **In control embryos, Pax6-expressing cells (purple) are located ventral to the developing internal capsule; those located laterally comprise the lateral cortical stream migrating from the pallial-subpallial border (PSPB). Islet1-expressing cells (green) migrate from the progenitor layer of the lateral ganglionic eminence (green arrow) to form the corridor through which thalamocortical axons grow (curved black arrow). **(B) **In conditional knockout (cKO) embryos, there are fewer Pax6-expressing cells ventral to the internal capsule than normal (other populations of Pax6-expressing cells outside the region of Cre-mediated *Pax6 *deletion are not shown since they are not affected). Cells from the lateral ganglionic eminence migrate to form a corridor that is abnormally broad with a lower peak density of Islet1-expressing cells; many Islet1-expressing cells stray into the area depleted of Pax6 expression. Many thalamic axons fail to enter this abnormal corridor or exit it along its length.

In the telencephalon, Pax6 is considered a major regulator of early dorsal patterning and development and its functions in ventral telencephalon, where it is less widely expressed, are obscure [27,42,46-49]. Our work indicates that ventral telencephalic Pax6 expression plays an important part in the development of the corridor that guides thalamic axons through the ventral telencephalon towards the cerebral cortex. Depletion of ventral telencephalic Pax6-expressing cells causes a diluted and broadened distribution of corridor cells and misrouting of some thalamic axons, both around their region of entry into the corridor and along its length. Our main findings are summarized in Figure 9.

### Generation of cKO embryos with Pax6 depletion restricted to cells ventral to the internal capsule

We have made a new floxed allele of the *Pax6 *gene in which none of the *loxP *elements reside in an exon or in any conserved sequence element with the aim of minimizing the chance of interfering with the normal expression of the gene in the pre-deleted state. This offers a different floxed allele to that generated by Ashery-Padan *et al. *[50] in which one *loxP *site is in exon 4 upstream of the first ATG site. Neither their nor our *Pax6*^*loxP/loxP *^mice show any obvious morphological defects.

Pax6 is normally expressed at several sites in embryonic ventral telencephalon. First, it is expressed in the progenitor cells of the LGE; its expression here is much lower than its expression on the dorsal side of the PSPB [19,27,32,33]. Second, it is expressed in neurons in the LCS that are derived from the vicinity of PSPB and migrate to occupy the basolateral complex [41]. Third, Pax6-expressing cells are located ventral to the developing internal capsule in the MGE; since these cells are present at E12.5, when the very first LCS cells are occupying the lateral part of the LGE, they might be derived from the progenitor zone of the MGE rather than the LCS. Using a *Six3*^*A1A2*^-*Cre *allele [39], we were able to obtain a highly specific depletion of Pax6-expressing cells ventral to the developing internal capsule at the age when thalamic axons first turn into it. Cre recombinase was expressed neither in the proliferative zone of the LGE, nor in the dorsal thalamus, nor in the cortex, and Pax6 continued to be expressed as normal in these regions.

### Comparison of thalamic axonal defects in *Pax6*^-/- ^and Pax6 cKO embryos

The thalamocortical tract does not form in *Pax6*^-/- ^mice and rats [12,19-21]. *Pax6*^-/- ^thalamic cells generate a large bundle of axons that grow at the same time as, have the same fasciculation pattern as, and extend initially along the same route as wild-type thalamocortical axons. They become misrouted as they cross into ventral telencephalon. Many extend aberrantly into the hypothalamus [12,21]. Others enter the internal capsule but then exit it to enter the future amygdala in the ventral part of the ventral telencephalon [21]; thalamic axons were also observed taking this route when grown into *Pax6*^-/- ^ventral telencephalic slices *in vitro *[20]. Others enter the ventral telencephalon abnormally ventrally, around the base of the telencephalon [12,19]. In the cKOs described here, we found evidence for less extreme versions of these defects: although many thalamic axons navigated through the internal capsule, others deviated ventrally towards the hypothalamus (Figure 5B"); some of those that had deviated ventrally towards the hypothalamus later projected laterally into the ventral part of the ventral telencephalon (green arrowheads in Figure 5C, D); some axons that did turn into the internal capsule then exited it in a ventral direction towards the amygdala (Figure 4E"). These findings indicate that at least some of the defects of thalamocortical navigation reported in *Pax6*^-/- ^embryos are caused by loss of Pax6 from ventral telencephalon.

It would be interesting to discover whether the degree of Pax6 depletion in the region ventral to the developing internal capsule influences the proportion of thalamic axons that navigate correctly. Testing this possibility was not possible here since the *Six3*^*A1A2*^-*Cre *allele produced a depletion that was highly consistent from embryo to embryo, as shown by the tight error bars on the data in Figure 2H, K, and so there was no opportunity to correlate a variable level of depletion with the phenotype. The development of a new Cre-expressing line in which an inducible form of Cre recombinase is expressed by all cells restricted to the tissue ventral to the internal capsule might allow this possibility to be tested, although finding a suitable promoter will be a challenge. Nevertheless, our data do show a clear requirement for normal Pax6 expression in this region for normal thalamic axonal navigation.

While it is conceivable that complete removal of Pax6 from the region ventral to the developing internal capsule might reproduce the *Pax6*^-/- ^phenotype in its entirety, previously published work indicates that the *Pax6*^-/- ^thalamic axonal phenotype is more likely to arise from a combination of thalamic and ventral telencephalic defects. Two studies have applied methods to examine the effects of loss of Pax6 selectively in cortex or thalamus on thalamocortical development. While recent work with mice carrying a cortex-specific deletion of Pax6 showed that Pax6 is not required in the cortex for thalamocortical formation [31], earlier work suggested that loss of Pax6 in thalamic cells prevents its axons from responding appropriately to ventral telencephalic guidance cues [20]. It seems most likely, therefore, that ventral telencephalic defects in the deployment of guidance cues combine with defects in the ability of thalamic axons to respond to any cues that may be present in embryos lacking Pax6 to give the full thalamic axonal phenotype of the *Pax6*^-/- ^embryo.

### Mechanisms of aberrant thalamocortical axonal navigation in *Pax6 *cKO embryos

The axons in cKOs that fail to turn through the internal capsule but stray ventrally, roughly parallel to the diencephalic-telencephalic border, follow a route taken not only by thalamic axons in *Pax6*^-/- ^mutants, but also by at least some thalamic axons in *Emx2*^-/- ^[13], *Mash1*^-/- ^[6], *Foxg1*^-/- ^[20] and *Lhx2*^-/- ^[51] mutants. It is possible that this is a default pathway taken by thalamic axons that fail to be attracted into the region of the future internal capsule. In these mutants, it has been suggested that the failure of attraction is due to defects of reciprocal projections from the region of the internal capsule that would normally act as guidance cues [8,14,51]. In the case of *Pax6*^-/- ^mutants the dorsal thalamus is not innervated by pioneering axons from ventral telencephalon [14]. Our present results show that, in normal embryos, this pioneering population does not express Pax6 at the time when it might provide guidance to thalamic axons, although we can not exclude the possibility that it is derived from cells that expressed Pax6 previously, and that it does still innervate the thalamus in cKOs with depletion of Pax6 only in ventral telencephalon. It is possible that in full *Pax6*^-/- ^mutants the absence of these pioneer projections occurs because thalamus, which normally expresses Pax6 and is defective, does not attract these axons, whereas in *Pax6 *cKOs the thalamus (which is normal) does attract the pioneer projections. Overall, our findings argue against the possibility that a ventral telencephalic function of Pax6 is to regulate the development of pioneer projections from ventral telencephalon to thalamus. They suggest that presence of these pioneer projections is not sufficient to ensure that all thalamic axons are routed normally.

We then looked for defects of the other major feature of the ventral telencephalon that guides thalamocortical axons, the Islet1-expressing corridor [15]. We found a diluted and broadened distribution of corridor cells at E12.5, the age at which thalamic axons are first turning towards this corridor region. The fact that the *Six3*^*A1A2*^-*Cre *allele did not remove Pax6 from the proliferative zone of the LGE (which expresses Pax6) is important since this is the region from which the Islet1-expressing corridor cells migrate (green arrows in Figure 9). We can conclude, therefore, that the cells destined for the corridor in the *Pax6 *cKOs were normal and that the cause of their abnormally broadened distribution into a ventral region that expresses Pax6 is almost certainly a result of the depletion of Pax6 in that region. The cells of the corridor migrate at around E12.5, by which stage Pax6 was already depleted in the mantle zone of the MGE in cKOs, implicating Pax6 in the regulation of the final distribution of the immigrant cells. It is possible that Pax6-expressing cells ventral to the corridor exert a repulsive influence on the incoming Islet1-expressing LGE-derived cells. The reason that many thalamic axons failed to enter the corridor in *Pax6 *cKOs might have been the reduced peak-densities of corridor cells. The reason that a proportion of the thalamic axons that did successfully enter the corridor and began to travel along it exited by taking a ventral turn might have been the abnormal ventral dispersal of cells that should have migrated into the corridor, thereby providing a more permissive environment for these axons in ventral tissue.

A fruitful focus for further work would be an analysis of the molecular mechanisms by which the transcription factor Pax6 normally constrains the migration of corridor cells. At present we do not understand how cells are guided from the LGE into the corridor but it is likely that differential cell-cell adhesion, allowing the migrating corridor cells to enter some regions but keeping them out of others, plays a critical role. Pax6 is known to regulate the expression of cell adhesion molecules and regulate cell migration elsewhere in the developing telencephalon [30,52,53], increasing the likelihood that its effects in the ventral telencephalon are mediated via its control over the expression of these types of molecule.

It is possible that depletion of Pax6 in ventral telencephalon might affect thalamic axonal navigation relatively directly through defective expression of one or more of a potentially large number of cell surface molecules that might affect the navigation of thalamic axons into and through the internal capsule (reviewed in [8]). Indeed, the effects of ventral Pax6 depletion on corridor and thalamic axonal development might be parallel results of the same molecular changes and defects of the corridor might not be the cause of thalamic axonal defects. Thus, while it is tempting to link causatively the defects of corridor development to those of thalamic axonal navigation in *Pax6 *cKOs, much more work will be needed to establish or refute this hypothesis.

### Defects of corticofugal axons in *Pax6 *cKOs

We found evidence that some corticofugal axons, specifically from the visual cortex, are misrouted and descend laterally in the telencephalon rather than entering the internal capsule. It is possible that this is caused by the defect in the population of ascending thalamocortical projections. Previously it has been suggested that the intermingling of ascending thalamocortical axons with descending corticofugal axons in the ventral telencephalon is important for the guidance of each set [3,4,8,9,21]. It seems highly plausible, therefore, that a diminished population of ascending thalamic axons might be unable to provide guidance for all descending cortical axons, causing some to deviate laterally. A possible reason for a specific effect on axons from the visual cortex is that these axons might arrive at the point of interaction with ascending fibers last, since they come from the caudal cortex that is further from the internal capsule and whose development lags behind that of more rostral cortex [54]. Their late arrival might disadvantage their ability to locate and/or interact with guidance partners in the diminished ascending population. Clearly, other explanations are possible: for example, Pax6 might be needed to regulate the expression of molecules around the internal capsule that attract visual corticofugal axons through this region. Whatever the cause, it can not be an autonomous effect in the corticofugal cells themselves since cortical expression of Pax6 is unaffected in these cKOs.

It is conceivable that in cKOs defects of the descending corticofugal axons, caused directly by misregulation of expression around the internal capsule, might contribute to the defects of the ascending thalamic axons. Previous studies have suggested that descending corticofugal axons, on crossing the PSPB at about E14.5, interact and help guide ascending thalamocortical axons across the PSPB [3,4,8,9,21]. This mechanism might contribute to defects of thalamic axons within the internal capsule; it seems a less likely explanation for early defects of thalamic axons around the diencephalic-telencephalic boundary.

## Conclusion

Our findings indicate an important novel function of Pax6 along the ventral aspect of the ventral telencephalon, constraining migrating LGE cells to form a tight Islet1-expressing cell-dense corridor and providing normal guidance cues for developing thalamocortical axons.

## Materials and methods

### *Pax6 *gene targeting

The RPCI-21 mouse PAC library [55] was screened with a *Pax6 *intron 6 probe and a clone, 450-I22, was cloned into the plasmid pZero2 (Invitrogen, Paisley, UK). A 10 kb intron 4 and 6 containing subclone, 247B1, was identified. A 1.8 kb *Bam*HI-*Sac*I fragment containing part of exon 4 and its downstream intron was subcloned from 247B1 and the *loxP *site-flanked neomycin resistance cassette from plasmid pNeoflox8 (W Muller, University of Cologne, Germany) was inserted into an *Afl*II site within a non-conserved region of mouse intron 4, generating a 3.1 kb insert. A 3.3 kb *Sac*I-*Bam*HI fragment containing exons 5, 5a and 6 and a 3.8 kb *Bam*HI-*Not*I fragment containing exon 7 were amplified from 129SvJ genomic DNA and cloned into pCR-BluntII-TOPO (Invitrogen). The reverse primer for the 3.3 kb fragment contained a single *loxP *site linked to a *Bam*HI site. The 3.1, 3.3 and 3.8 kb fragments were ligated together by oriented cloning to produce the final 10.2 kb targeting fragment (Figure 1A). The *Not*I linearized targeting construct (50 μg) was electroporated into E14Tg2a embryonic stem (ES) cells and neomycin resistant cell clones isolated after 10 days in culture. Clones were screened by Southern blot to identify those that had undergone homologous recombination (Figure 1B, C). Chimeric mice were generated by injecting C57BL/6 blastocysts with ES cells derived from three independent ES clones (4-B4, 6-B5 and 6-F9) of normal karyotype. F1 animals were genotyped by PCR with the forward primer 5'-AAATGGGGGTGAAGTGTGAG-3' and reverse primer 5'-TGCATGTTGCCTGAAAGAAG-3' that flank the single *loxP *site (Figure 1D) to identify founders.

### Mouse mutants and breeding

The floxed *Pax6 *allele (designated *Pax6*^*tm1Ued *^using Mouse Genome Informatics nomenclature) is referred to as *Pax6*^*loxP *^and the floxed-deleted allele as *Pax6*^*loxPΔ*^. Lines of mice carrying this allele and other transgenes (*Six3*^*A1A2*^-*Cre *[39]; *Zp3-Cre *[35]; *Z/AP *reporter [40]) were back-crossed for at least six generations to the Crl:CD-1 (ICR; Charles River, Tranent, UK) strain and were subsequently maintained on that background. The age of each embryo was counted from the morning of the vaginal plug (deemed E0.5) and confirmed by morphology. To generate conditional homozygous knockout animals (designated cKO for convenience), males either homozygous for the floxed *Pax6 *allele or compound homozygous for the floxed *Pax6 *and *Z/AP *alleles were crossed with females compound heterozygous for the floxed *Pax6 *allele and either the *Six3*^*A1A2*^-*Cre *allele or the *Zp3-Cre *allele. These crosses also generated embryos that were used as controls, that is, homozygous for the floxed *Pax6 *allele but lacking the *Six3*^*A1A2*^-*Cre *allele or compound heterozygous for the floxed *Pax6 *allele and the *Six3*^*A1A2*^-*Cre *allele. Neither two copies of the floxed-undeleted allele nor a single copy of a deleted allele caused detectable telencephalic defects. Animal care followed institutional guidelines and UK Home Office regulations.

### Genotyping by PCR

Mice carrying the *Pax6*^*loxP *^allele were genotyped using primers described above (Figure 1A, D). Mice carrying the *Pax6*^*loxPΔ *^allele were genotyped using a forward primer in intron 4 (5'-TTACCCTGGCTTTGCTTTTG-3') and a reverse primer in intron 6 (5'-GGAGCAGTCCTTCACCTCTG-3') downstream of the single distal *loxP *site (Figure 1A, D). Cre recombinase-expressing transgenic mice were genotyped using primers to the *Cre *cassette (forward 5'-CATTTGGGCCAGCTAAACAT-3', reverse 5'-ATTCTCCCACCGTCAGTACG-3'). *Z/AP *transgenic mice were genotyped using primers to the hPLAP-encoding cassette (forward 5'-AACCCCAGACCCTGAGTACC-3', reverse 5'-GTGGAGTCTCGGTGGATCTC-3'). PCR reactions used standard conditions.

### Histology

Mouse embryos were dissected in ice-cold phosphate-buffered saline (PBS) and then fixed by shaking in 4% (w/v) paraformaldehyde (PFA) in PBS at 4°C overnight. Following fixation, embryos were dehydrated, embedded in paraffin wax, sectioned at 10 μm in the coronal plane and mounted on poly-L-lysine coated slides.

### Immunohistochemistry

Sections were dewaxed in xylene and hydrated through alcohols (including a 15 minute incubation in 3% (v/v) H_2_O_2 _in methanol to aid epitope recovery) to PBS, then boiled in 10 mM sodium citrate (pH 6) in a microwave. After blocking in 10% normal goat serum in PBS with 0.1% (v/v) Triton X-100 (PBS-TX), sections were incubated with primary antibodies at 4°C overnight. Sections were then washed twice in PBS-TX and incubated in 10% normal goat serum in PBS-TX for 10 minutes. Sections were incubated in a 1:200 dilution of biotin-conjugated goat anti-mouse secondary antibody in 10% normal goat serum in PBS-TX for 1 hour at room temperature and rinsed again in PBS-TX. An avidin-biotin reaction was carried out using 0.05% (w/v) diaminobenzidine in tris-buffered saline containing 0.02% H_2_O_2_. Sections were rinsed in water, dehydrated, cleared in xylene and mounted. Primary antibodies were for Pax6 (1:40; AD2.38, a gift from Professor V van Heyningen, MRC Human Genetics Unit, Edinburgh, UK), Islet1 (1:200; DSHB, Iowa City, IA, USA) and Mash1 (1:100; BD Pharmingen, San Jose, CA, USA).

### Alkaline phosphatase staining

To reveal hPLAP activity, embryos were first dissected in ice cold PBS. Brains from embryos older than E12.5 were removed from their skulls and bisected parasagittally prior to fixation for 0.5 to 2 hours in 4% PFA on ice on a shaking platform. Tissue was then rinsed in ice cold PBS and embedded in 4% (w/v) agarose in PBS in blocks. Blocks were sectioned coronally on a vibratome at 100 to 200 μm. Sections were collected into wells containing PBS and then stained for hPLAP activity as described previously [40]. Sections were post-fixed in 2% (v/v) glutaraldehyde in PBS for 2 hours at 4°C, rinsed several times in PBS, cleared by passing through 1:1 (w/v) and 9:1 (w/v) glycerol:PBS and then mounted in 9:1 glycerol:PBS.

### Carbocyanine dye injection and analysis

Brains were fixed overnight by shaking in 4% (w/v) PFA at 4°C. Two different methods were used to label the thalamocortical tract. In the first, whole brains were dissected away from their skulls and a medial slice of cortex was removed on both sides of the midline to expose the dorsal surface of the thalamus. Single crystals of the lipophilic tracer DiI were injected at three symmetrical positions along the rostrocaudal extent of the thalamus. In the second, whole brains were bisected parasagittally to expose the dorsoventral aspect of the thalamus at the midline. Injections of single crystals were made at three positions along the dorsoventral extent of the dorsal thalamus. In some experiments injections of DiI and the lipophilic tracer DiA were made in the cortex. All injections were made by picking up single DiI crystals with pulled glass capillaries and lancing the tissue at each desired location to deposit the crystal. Dyes were allowed to diffuse at room temperature for 4 to 6 weeks in 4% (w/v) PFA in PBS, rinsed in PBS, embedded in agarose, sectioned coronally on a vibratome at 100 to 200 μm, counterstained with 0.002% (w/v) bisbenzimide in PBS for 30 minutes at room temperature and cleared through glycerol.

### Microscopy

All images were acquired using an epifluorescence microscope mounted with a digital camera. In epifluorescence, bisbenzimide appears blue (UV filter) and DiI appears red/orange (rhodamine filter).

## Abbreviations

cKO: conditional knockout; E: embryonic day; ES: embryonic stem; hPLAP: human placental alkaline phosphatase; LCS: lateral cortical stream; LGE: lateral ganglionic eminence; MGE: medial ganglionic eminence; PBS: phosphate-buffered saline; PBS-TX: PBS with 0.1% (v/v) Triton X-100; PFA: paraformaldehyde; PSPB: pallial-subpallial border.

## Competing interests

The authors declare that they have no competing interests.

## Authors' contributions

TIS generated the floxed allele and analyzed the crosses with Cre-expressing lines, TP contributed data on the expression of Pax6 in DiI-labeled brains, JOM and DJP supervised the work, and all authors contributed to the preparation of the final manuscript.

## Supplementary Material

Additional file 1 ***Pax6 *allele comparison at E12.5**. **(A-D) **Morphology of the telencephalon of (A) wild-type, (B) *Pax6*^*loxP/loxP*^, (C) *Pax6*^*loxPΔ/loxPΔ *^and (D) *Pax6*^*SeyEd/SeyEd *^mice compared using haematoxylin and eosin stained sections. There were no obvious structural differences between wild-type mice and mice carrying two copies of the floxed *Pax6 *allele. Mice carrying two copies of the *Pax6*^*loxPΔ *^allele shared the same phenotype as mice carrying two copies of the commonly studied *Pax6*^*SeyEd *^allele. (A', A", B', B") Pax6 expression, determined by immunohistochemistry, was indistinguishable between wild-type and *Pax6*^*loxP/loxP *^mice. (C', C") There was no detectable Pax6 protein in *Pax6*^*loxPΔ/loxPΔ *^mice. (D', D") Some residual Pax6 protein was detected in *Pax6*^*SeyEd/SeyEd *^mice. Scale bars: 50 μm. Click here for file

Additional file 2 **Normal expression of Pax6 in cortex and thalamus of cKOs at E14.5**. **(A, B) **Immunohistochemistry for Pax6 on coronal sections of cortex (Cx) from control and cKO embryos. **(C, D) **Immunohistochemistry for Pax6 on coronal sections of dorsal thalamus (DT) and ventral thalamus (VT) from control and cKO embryos. Scale bars: 50 μm. Click here for file
