# Supplementary material for: Normal ventral telencephalic expression of Pax6 is required for normal development of thalamocortical axons in embryonic mice
Source: Neural Dev. 2009 Jun 5;4:19. doi: 10.1186/1749-8104-4-19 (PMC2699344; doi:10.1186/1749-8104-4-19)
Supplement: Additional file 1 — Pax6 allele comparison at E12.5. (A-D) Morphology of the telencephalon of (A) wild-type, (B) Pax6loxP/loxP, (C) Pax6loxPΔ/loxPΔ and (D) Pax6SeyEd/SeyEd mice compared using haematoxylin and eosin stained sections. There were no obvious structural differences between wild-type mice and mice carrying two copies of the floxed Pax6 allele. Mice carrying two copies of the Pax6loxPΔ allele shared the same phenotype as mice carrying two copies of the commonly studied Pax6SeyEd allele. (A', A", B', B") Pax6 expression, determined by immunohistochemistry, was indistinguishable between wild-type and Pax6loxP/loxP mice. (C', C") There was no detectable Pax6 protein in Pax6loxPΔ/loxPΔ mice. (D', D") Some residual Pax6 protein was detected in Pax6SeyEd/SeyEd mice. Scale bars: 50 μm. [file 1749-8104-4-19-S1.pdf]

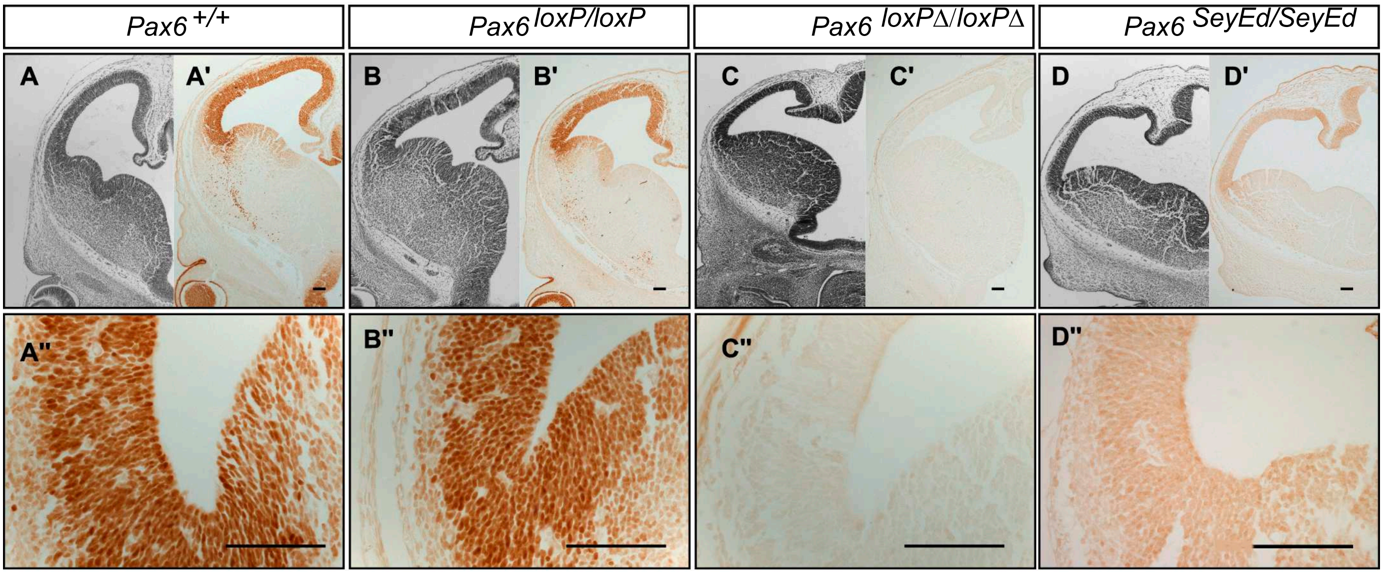

Supplementary Fig. 1. *Pax6* allele comparison at E12.5. (A-D) Morphology of the telencephalon of (A) wild-type, (B) *Pax6*$^{loxP/loxP}$, (C) *Pax6*$^{loxP\Delta/loxP\Delta}$ and (D) *Pax6*$^{SeyEd/SeyEd}$ mice compared using haematoxylin and eosin stained sections. There were no obvious structural differences between wild-type mice and mice carrying two copies of the floxed *Pax6* allele. Mice carrying two copies of the *Pax6*$^{loxP\Delta}$ allele shared the same phenotype as mice carrying two copies of the commonly studied *Pax6*^{SeyEd} allele. (A',A",B',B") Pax6 expression, determined by immunohistochemistry, was indistinguishable between wild-type and *Pax6*$^{loxP/loxP}$ mice. (C',C") There was no detectable Pax6 protein in *Pax6*$^{loxP\Delta/loxP\Delta}$ mice. (D',D") Some residual Pax6 protein was detected in *Pax6*$^{SeyEd/SeyEd}$ mice. Scale bars: 50µm.
