# Supplementary material for: Normal ventral telencephalic expression of Pax6 is required for normal development of thalamocortical axons in embryonic mice
Source: Neural Dev. 2009 Jun 5;4:19. doi: 10.1186/1749-8104-4-19 (PMC2699344; doi:10.1186/1749-8104-4-19)
Supplement: Additional file 2 — Normal expression of Pax6 in cortex and thalamus of cKOs at E14.5. (A, B) Immunohistochemistry for Pax6 on coronal sections of cortex (Cx) from control and cKO embryos. (C, D) Immunohistochemistry for Pax6 on coronal sections of dorsal thalamus (DT) and ventral thalamus (VT) from control and cKO embryos. Scale bars: 50 μm. [file 1749-8104-4-19-S2.pdf]

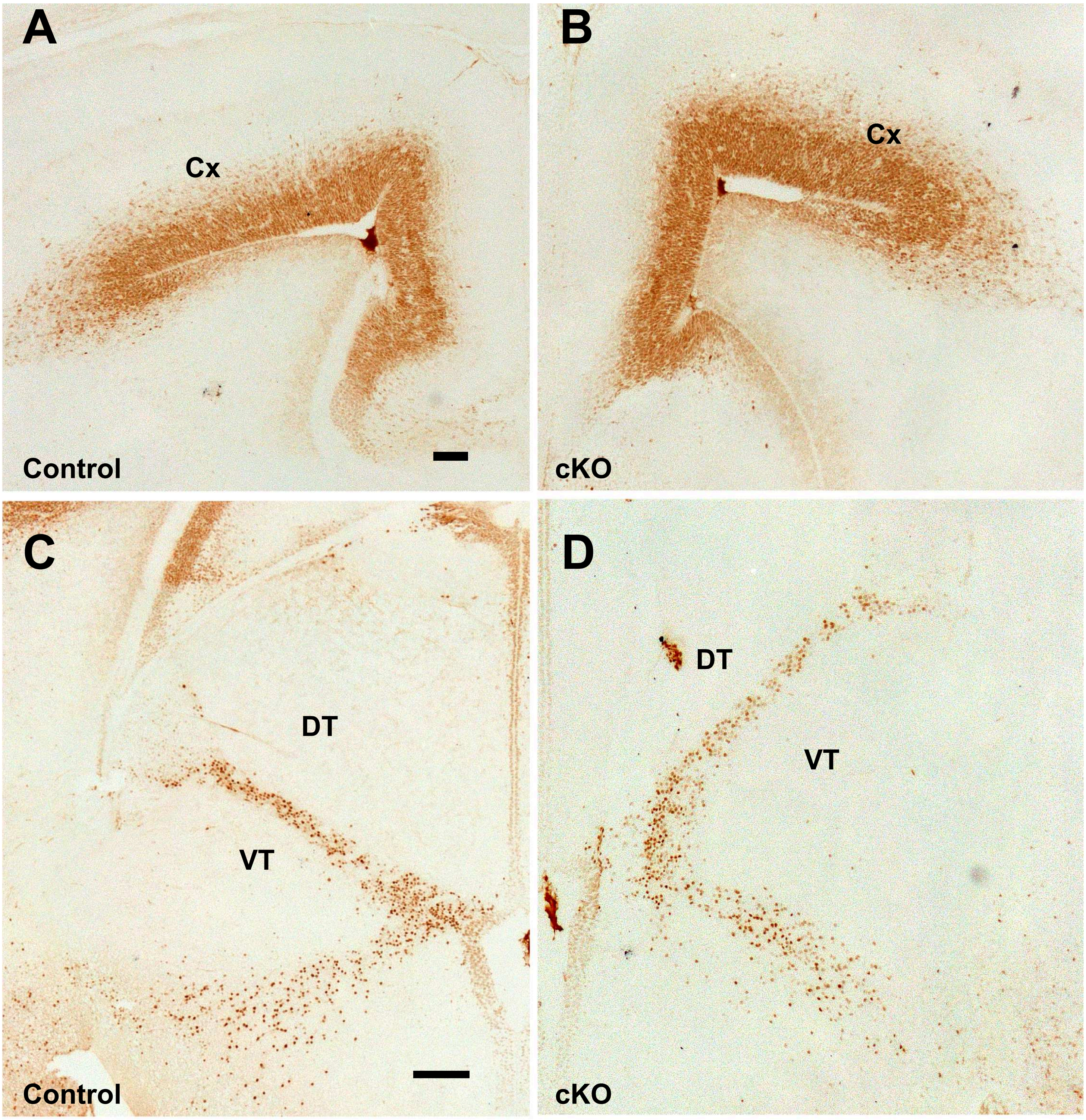

Supplementary Fig. 2. Normal expression of Pax6 in cortex and thalamus of cKOs at E14.5. (A,B) Immunohistochemistry for Pax6 on coronal sections of cortex (Cx) from control and cKO embryos. (C,D) Immunohistochemistry for Pax6 on coronal sections of dorsal thalamus (DT) and ventral thalamus (VT) from control and cKO embryos. Scale bars: 50µm.
